# Assessment of golden proportion and gingival aesthetic line classification in maxillary anterior teeth: A cross-sectional digital photographic analysis

**DOI:** 10.6026/973206300222496

**Published:** 2026-04-30

**Authors:** Shouq Abdulrahman AlMathami, Abdulrahman Ahmed Ismail, Mohammed Ghazi Sghaireen, Doaa Abdelaziz A. Helal

**Affiliations:** 1 Department of Prosthetic Dentistry, College of Dentistry, Jouf University, Sakaka 72345, Aljouf, Saudi Arabia; 2 Department of Fixed Prosthodontics, Beni-Suef University, Egypt

**Keywords:** Golden proportion, gingival aesthetic line, gingival zenith, maxillary anterior teeth, dental aesthetics, digital photographic analysis

## Abstract

The applicability of the golden proportion and gingival aesthetic line harmony in natural anterior dentitions remains controversial across different populations. Therefore, it is of interest to evaluate the presence of the golden proportion and gingival aesthetic line characteristics in maxillary anterior teeth among 200 adults using standardized digital photographic analysis. Mesiodistal tooth width ratios were calculated and compared with the theoretical golden ratio (0.618), while gingival zenith positions and gingival line patterns were also assessed. The results showed that both lateral-to-central incisor and canine-to-lateral incisor ratios significantly deviated from the golden proportion, whereas Class I gingival aesthetic line patterns were more consistently observed. Thus, we show that natural anterior aesthetics are not governed by strict mathematical proportions, emphasizing the importance of individualized treatment planning over formula-based approaches.

## Background:

The way of contemporary aesthetic dentistry has been transformed radically since it has been the field where the primary goal was to restore the functions of the piece, whereas now it is the field that incorporates biological laws, mathematical relations and aesthetics to obtain the final result that would be in harmony with the overall composition of the face [1]. The maxillary dentition that is positioned in the front of the mouth performs the role of paramount significance in the field of smile aesthetics and it is the centre of interpersonal communication and social perception. As such, there has been significant scholarly attention on setting objective parameters that may describe perfect anterior tooth proportions, symmetry and gingival design [2]. The golden proportion is one of the mathematical ideas that have been used in dental aesthetics that has received the most sustained attention [3]. The golden ratio, which is also called the divine ratio, is a mathematical proportion of about 1:1.618 or 0.618:1 that has been observed in many natural and art forms of the past. The original implementation of this notion into dentistry suggested that the apparent mesio-distal breadth of every maxillary anterior tooth, regarded in the frontal perspective must be about 61.8 percent of the breadth of the adjacent more mesially located tooth [4]. By this model, the perceived width of the lateral incisor is supposed to be 61.8 percent of the central incisor width and the perceived canine width is also supposed to be 61.8 percent of the lateral incisor width. The following mathematical expression was postulated to be used as a guide towards achieving natural-feeling proportionality during the development of prosthetic and restorative dentistry interventions [5]. Nonetheless, many studies have been done on the golden proportion of natural dentitions with the aim of questioning the validity of the ratio in clinical practice because of its uncertainty in various ethnic and demographic groups of the population. A number of population studies have shown that a small portion of naturally attractive smiling faces follow the golden ratio strictly and that the golden ratio is only theoretical in theory and not biologically feasible [6]. As a reaction to these observations, alternative proportional systems have been put forward, such as the recurring aesthetic dental proportion, that a regular percentage decrease in apparent tooth width ought to transpire when relocating backwards on the midline, but that percentage need not be precisely 62 percent [7]. In addition to the aspect of proportionality of width of teeth, the architecture of gingival tissues also has a significant effect on the perception of the aesthetic of the anterior smile. The apparent length, axial inclination and symmetry of individual teeth in the dental arch are determined by the gingival zenith that is the highest point of the free gingival margin on the labial side of a tooth [8]. Gingival zenith position deviations may produce aesthetic effects that are highly visualized and thus severely affect aesthetic results despite having tooth dimensions within acceptable levels [9]. The gingival aesthetic line is a clinical reference framework that has been designed to systematically compare the association between the gingival zenith positions of the maxillary anterior teeth. The construction is based on the linking of the gingival zeniths of the central incisor and the canine on either side or the point where the lateral incisor zenith is in relation to this line will make the classification. A classification system has been suggested whereby Class I is suggested to have GAL angle (45-90 Degree) and the lateral incisor zenith slightly coronal by 1-2mm or touching the gingival aesthetic line, Class II suggested to have GAL angle (45-90 Degree) but the lateral incisor located apically 1-2mm to the zeniths with mesial part overlaps the distal part of central incisor, Class III suggests to have GAL angle (= 90 degree) and the zeniths of central, lateral and canine are all in the same level and Class IV represents those patterns that are not in any of the standard classification [10]. The gingival line angle is defined as the angle between the gingival aesthetic line and a vertical reference line point at the interdental papilla at the midline gives further quantitative data on gingival line symmetry [11]. Although there is significant amount of literature on either tooth proportions or gingival contour features as independent variables, very few studies have concomitantly measured both the golden proportion and gingival aesthetic line within the same sample group through standardized photographic measures. This combined evaluation is of clinical significance, in that aesthetic perception is a compound phenomenon that is determined by the engagement of the dental and gingival parameters as opposed to each constituent individually [12]. Moreover, digital photographic examination has become a valid and reproducible way of assessing visible dental and gingival sizes, as seen in the frontal view, which is the clinically useful viewpoint in which aesthetic judgments are created [13]. Therefore, it is of interest to evaluate the prevalence of the golden proportion and describe gingival aesthetic line patterns in maxillary anterior teeth using standardized digital photographic analysis.

## Materials and Methods:

## Study design and ethical issues:

The study was a cross-sectional observational study of the adult participants who attended the outpatient clinical department at College of Dentistry, jouf University, Saudi Arabia within a span of eight months. The institutional ethics committee agreed and approved (No. HAPO-13-S-001 \ 11436) the study protocol prior to the start of data collection. All the participants provided informed consent in writing that was well explained to them about the study objectives and procedures. It carried out its research following the principles of the Declaration of Helsinki concerning research using human subjects.

## Sample size determination:

The estimation of sample size was done based on the single proportion formula of finite population correction. A small pilot data on the projected prevalence of golden proportion adherence of about 25 percent, desired precision of 8 percent, a 95 percent confidence level and an 80 percent power confirmed a small sample size 113 participants. Considering possible exclusions and incomplete photographic records, the sample size was increased to 200 participants. The final sample therefore consisted of 200 individuals, with equal gender distribution (100 males and 100 females).

## Participant selection:

The participants were chosen by simple continuous convenience sampling of people that visit their dentists regularly. The inclusion criteria were as follows: Age was 20-40 years old, full eruption of intact maxillary anterior teeth (canine to canine), no dental restorations or prosthetic crowns in the anterior region, clinically healthy periodontal tissues with probing depth of not more than 3 millimetres, no history of orthodontic treatment and no apparent spacing, rotation, or crowding of the anterior part. Exclusion criteria were; presence of gingival recession on one of the maxillary anterior teeth, attrition or abrasion of incisal ridge or change in clinical crown morphology, prior aesthetic dental procedure such as bleaching or veneers, craniofacial anomaly or developmental dental anomaly and occurrence of gingival enlargement or inflammation that would interfere with the identification of zenith of gingival ridge.

## Photographic protocol:

All the participants were taken to have standardized frontal intraoral photographs taken by a Digital Single Lens Reflex (DSLR) camera (Nikon D5600, Nikon Corporation, Tokyo, Japan) equipped with a 60-mm Macro-Lens (AF-S Micro-NIKOR 60 mm) and Macro-Ring Flash (Godox MF-R76) to ensure standardized illumination. A standard camera setting was used (Shutter speed 1\125, ISO 200, Aperture: 10). The camera was positioned using a tripod at the level of the maxillary anterior teeth, with the optical axis perpendicular to the facial midline and with distance 1 Meter away from participant. Polytractor (Cheek Retractor) were used to displace and stabilize the lip and cheeks during intra- oral photograph. Each photograph was followed by placing a calibration reference scale on the adjacent area of the anterior teeth which was a 10-millimeter graduated ruler in the same focal plane. This reference allowed the appropriate conversion of pixel values into millimetre values in the course of the analysis. Photographs were acquired by a single operator to reduce inter-operator variability and images were checked in terms of sufficient focus, exposure and all six anterior teeth after which they were accepted.

## Image measures and analysis:

Digital measurements were performed using Adobe Photoshop 2026 (Adobe Systems Inc., San Jose, CA, USA). The software calibration tool was used to convert pixel measurements into millimetres using the reference scale. Measurement was made by one trained examiner and a random sample of 30 images was re-measured after a two weeks period to assess intra-examiner reliability that resulted in an intraclass correlation coefficient value of over 0.92 across all parameters measured. The classification was determined separately for the right and left sides of each participant. When both sides exhibited the same class, the participant was assigned that class. In cases where the right and left sides showed different classifications, the predominant pattern was recorded for statistical analysis.

## Golden proportion evaluation:

The perceived mesio-distal width of individual maxillary anterior teeth was quantified as a maximum horizontal distance between the mesial and distal contacts points as viewed in the frontal view. Each individual was measured in three teeth; the central incisor (designated as dimension A), the lateral incisor (designated as dimension B) and the canine (designated as dimension C).

The golden proportion ratios were obtained by means of the following formulas:

[1] Lateral incisor to central incisor ratio: B/A

[2] Canine to lateral incisor ratio: C/B

These ratios have been computed to the right and the left sides. A ratio was taken to agree with the golden proportion when the ratio lies between 0.598 and 0.638, which was equated to a tolerance of 0.02 with the theoretical value being 0.618.

## Evaluation of gingival aesthetic line classification:

It was determined that the apical point of the labial surface of the free gingival margin of every maxillary anterior tooth was the gingival zenith. A line was drawn digitally between the gingival zenith of the central incisor and gingival zenith of the canine on each side termed as the gingival aesthetic line. The distance of the gingival zenith of the lateral incisor to this line was perpendicularly measured vertically.

Under this measure, each side was identified under the following classification:

[1] Class I: The GAL angle is between 45°C and 90°C and the lateral incisor is touching or below (1- 2 mm) the GAL

[2] Class II: The GAL angle is between 45°C and 90°C but the lateral incisor is above (1-2 mm) the GAL and its mesial part overlaps the distal aspect of the central incisor. This situation is often seen in Angle's Class II or pseudo-Class II conditions and adds variety to the dental composition

[3] Class III: The GAL angle = 90°C and the canine, lateral and central incisors all lie below the GAL

[4] Class IV: The gingival contour cannot be assigned to any of the above three classes

The GAL angle can be acute or obtuse. Myriad gingival asymmetries are apparent clinically including: recession, passive and altered passive eruption, eccentric eruption patterns, loss of interdental papillae, clefts and high frenal insertions.

## Statistical analysis:

All the data were keyed into a spreadsheet and were analysed with the help of the Statistical Package of Social Sciences software version 26. Continuous were indicated in the form of mean and standard deviation and categorical indicated in the form of frequencies and percentages. Normality of continuous variables was assessed by using the Shapiro-Wilk test. The one-sample t-test was used to make a comparison between the observed values of B/A and C/B ratios and the golden proportion value of 0.618. First, right-side and left-side ratios and gingival line angles were tested based on paired-samples t-tests. The differences between the distributions of the gingival aesthetic line classifications were assessed using chi-square test. All analyses were regarded as significant with a p-value that was below 0.05 at a two-tailed level.

## Results:

A total of 200 participants were included in the final analysis, with equal distribution of males (n = 100, 50%) and females (n = 100, 50%). The mean age of the study cohort was 23.4 ± 3.1 years, with a range of 20 to 40 years. All participants met the inclusion criteria and provided complete photographic data suitable for analysis. The mean mesio-distal width ratios for lateral incisor to central incisor (B/A) and canine to lateral incisor (C/B) are presented in Table 1 (see PDF). On both sides, the mean B/A ratios were significantly higher than the theoretical golden proportion value of 0.618, indicating that lateral incisors were proportionally wider relative to central incisors than predicted by the golden ratio. Conversely, the mean C/B ratios were slightly less than 0.618 on both sides, indicating that canines were proportionally narrower relative to lateral incisors than the golden proportion would predict. All four comparisons reached statistical significance. When the tolerance range of 0.598 to 0.638 was applied, strict adherence to the golden proportion was observed in 56 participants (28.0%) for the B/A ratio and 48 participants (24.0%) for the C/B ratio. Only 24 participants (12.0%) demonstrated golden proportion adherence simultaneously for both ratios on at least one side. Paired-samples t-tests comparing right and left sides revealed no statistically significant differences for either the B/A ratio (p = 0.287) or the C/B ratio (p = 0.341), indicating acceptable bilateral symmetry in tooth width proportions. The distribution of gingival aesthetic line classifications across the 200 participants is presented in Table 2 (see PDF). The mean gingival line angles for the left and right sides are presented in Table 3 (see PDF). The mean gingival line angle on the left side was 68.4 ± 9.2 degrees, while on the right side it was 69.1 ± 8.8 degrees. The paired-samples t-test revealed no statistically significant difference between sides (p = 0.412), indicating bilateral symmetry in gingival line angle distribution. The majority of participants (n = 148, 74.0%) demonstrated gingival line angles within the range of 55 to 80 degrees bilaterally. Angles below 45 degrees were observed in 12 participants (6.0%), while angles exceeding 85 degrees were observed in 18 participants (9.0%).

## Discussion:

The current study offers simultaneous data on the incidence of golden proportion compliance and gingival aesthetic line features on the maxillary anterior dentition of a population sample of young adults who had had no tooth restorations. These results support the idea that adherence to the golden proportion is rarely seen in natural dentitions and the gingival aesthetic line classification shows some consistent trends which can be more useful clinically to aesthetic harmony. The fact that the mean lateral to central incisor width ratio is greater than suggested golden proportion value is in line with extensive preceding studies. Initial studies of applicability of the golden proportion to natural dentitions had determined that although an idea was indeed attractive intellectually, often measurements had shown the existence of ratios quite far off the mathematical ideal [14]. The current result that 28.3 percent of respondents were able to be in the golden proportion in case of the B/A ratio is similar to the prevalence of the research undertaken in various populations with an average prevalence of 17-35 percent [15]. This uniformity in different study samples indicates that the golden proportion is an aesthetic ideal that is not a true reflection of the proportions in most of the natural dentitions. The C/B a ratio actually being slightly lower than expected in this research shows that the observed canine width was not as big as it should have been in accordance with the golden proportion, relative to the canine width and length. This observation can be a result of the normal arch shape which results in a gradual narrowing of tooth widths in the face view especially the ones that are found further laterally along the arch [16]. The extent of foreshortening will depend on the arch form, tooth rotation and viewing angle, which bring in variability that cannot be represented in a single mathematical constant. The fact that the bilateral differences between the width ratios are statistically insignificant is also a significant fact which proves the relative symmetry of the natural anterior dentition, although the proportions of individual teeth may not follow theoretical ideals. The bilateral symmetry in the proportions of teeth has been frequently reported in the literature and it has been suggested to be a stronger feature of a natural dentition than its compliance with a particular proportional formula [17]. This finding has significant clinical implications because it indicates that the bilateral symmetry can play a more significant role in determining aesthetic success than a specific target ratio. Recurring aesthetic dental proportion is a more flexible concept than the rigid golden proportion developed to propose that the successive ratio of perceived tooth widths may vary between 60 and 80 percent instead of being fixed at 62 percent [18]. The current data have a general agreement with this more lenient system in that the ratios that were observed were within the range indicated by this alternative proportional system in a greater number of participants compared to those who were within the rigid golden proportion measure. The trend with the Class I pattern of gingival aesthetic line identified in this study concurs with the past studies which have been used to assess the relationship of the gingival zenith in the natural dentitions. The most common arrangement is reported to be the Class I pattern where the lateral incisor gingival zenith lies slightly coronal to the line between the central incisor and canine zeniths and is reported most common in the groups of people with healthy periodontal tissues and intact anterior dentitions [19]. This design produces a scalloped gingival which is often perceived to be aesthetically appealing and is believed to be an objective setup during aesthetic periodontal and restorative surgeries [20]. A relatively large percentage of Class II patterns (26.7 percent) suggest that there is a significant minority group who have lateral incisor gingival zeniths at an apical position with respect to the gingival aesthetic line. Although such arrangement has been related with poorer aesthetic perception in certain tests, it is worth noting that this arrangement is a normal anatomical variation, not pathological condition [21]. This difference can be important to the clinician because individually assessing the patient should be done instead of using one gingivally contour template. The results of the gingival line angle indicate a stable angularity between the gingival aesthetic line and the midline reference and most of the participants have angles within a rather small functional range. The angle symmetry in the gingival lines seen on both sides of this study confirms the fact that gingival architecture as is seen in the case of tooth proportionality is usually symmetrical in healthy individuals with healthy periodontium and aligned teeth [22]. Previous clinical studies have suggested that harmonious anterior gingival architecture is generally associated with gingival line angles ranging between 45°C and 85°C [23]. The fact that the dental proportionality and the gingival contours examination are used in the same study group can be discussed as a methodological strength and gives an opportunity to obtain a more detailed picture of the anterior aesthetic parameters. Smile aesthetics is a multivariate aspect where tooth size, gingiva structure, lip movements and facial ratios play the interrelationship in producing the overall aesthetic perception [24]. An examination of these elements separately can give us an incomplete investigation of aesthetic harmony. As it is applied in this research, digital photographic analysis has the benefits of reproducibility and standardization but has some limitations. Apparent mesio-distal widths calculated using two-dimensional photographs may not necessarily be accurate representations of true tooth dimensions measured using dental casts and digital callipers, because photographic measurements are the projected width at a particular viewing angle and are affected by arch curvature and lens distortion [25]. Nevertheless, this outward appearance size is the clinically important size, since aesthetic evaluations are done under the frontal perspective of viewing. This study is cross-sectional and makes it impossible to prove causal relationships between proportional characteristics and aesthetic perception. Also, the sample size restrains the generalization of the results to older age groups where the gingival recession, attrition or aging could not only change the proportions of teeth but also the architecture of the gum. The fact that only well-aligned dentitions are used to be excluded to achieve standardized measurement implies that the results are only applicable to well-aligned dentitions but not necessarily the rest of the population. It is recommended that future studies should also include the subjective aesthetic assessment both by dental and lay person to establish the proportional and gingival parameters that have the strongest impact on the perceived attractiveness. There would also be longitudinal studies that explore the changes on these parameters in relation to aging that would provide the valuable information. Digital scanning technology could offer three-dimensional analysis, which is more accurate and rises beyond certain limitations of analysis in two dimensions, on the basis of photographs [26].

## Conclusion:

We report that the golden proportion is not a reliable predictor of maxillary anterior tooth width relationships, with strict adherence observed in less than one-third of the sample. The lateral-to-central incisor ratio was generally higher, while the canine-to-lateral incisor ratio was slightly lower than the theoretical value, suggesting that natural dentitions do not conform to a single mathematical constant. In contrast, gingival aesthetic harmony, particularly the Class I gingival zenith pattern, demonstrated greater consistency along with bilateral symmetry of gingival line angles. Thus, we show that gingival contour plays a more stable role in anterior aesthetic perception than rigid mathematical tooth proportions. An individualized approach to tooth proportions and gingival architecture should therefore be prioritized in aesthetic dental treatment planning.
